# See How They Grow: Testing the feasibility of a mobile app to support parents’ understanding of child growth charts

**DOI:** 10.1371/journal.pone.0246045

**Published:** 2021-02-19

**Authors:** Gayl Humphrey, Rosie Dobson, Varsha Parag, Marion Hiemstra, Stephen Howie, Samantha Marsh, Susan Morton, Dylan Mordaunt, Angela Wadham, Chris Bullen

**Affiliations:** 1 National Institute for Health Innovation, Faculty of Medical and Health Science, University of Auckland, Auckland, New Zealand; 2 Plunket, National Education, Auckland, New Zealand; 3 Department of Paediatrics, Child and Youth Health, Faculty of Medical and Health Science, University of Auckland, Auckland, New Zealand; 4 Growing Up in New Zealand, Faculty of Medical and Health Science, University of Auckland, Auckland, New Zealand; 5 University of Adelaide, South Australia, Australia; 6 Flinders University, South Australia, Australia; ESIC Medical College & PGIMSR, INDIA

## Abstract

**Background:**

Mobile devices provide new opportunities for the prevention of overweight and obesity in children. We aimed to co-create and test an app that offered comprehensible feedback to parents on their child’s growth and delivered a suite of age-specific information about nutrition and activity.

**Methods:**

A two-phased approach was used to co-create the digital growth tool—See How They Grow—and test its feasibility. Phase one used focus groups (parents and professionals such as paediatricians and midwives) and a national on-line survey to gather requirements and build the app. Phase two involved testing the app over 12-weeks, with parents or carers of children aged ≤ 2-years. All research activities were undertaken exclusively through the app, and participants were recruited using social media and hard copy materials given to patents at a child health visit.

**Findings:**

Four focus groups and 101 responses to the national survey informed the features and functions to include in the final app. Two hundred and twenty-five participants downloaded the app, resulting in 208 eligible participants. Non-Māori/Non-Pacific (78%) and Māori (14%) had the highest downloads. Fifty-four per cent of participants were parents of children under 6-months. These participants were more likely to regularly use the app than those with children older than 6-months (64% vs 36%, *P = 0*.*011)*. Over half of the participants entered three measures (n = 101, 48%). Of those that completed the follow-up survey (n = 101, 48%), 72 reported that the app helped them better understand how to interpret growth charts.

**Conclusion:**

The app was acceptable and with minor modifications, has the potential to be an effective tool to support parents understanding of growth trajectories for their children. A larger trial is needed to evaluate if the app can have a measurable impact on increasing knowledge and behaviour, and therefore on preventing childhood overweight and obesity.

## Introduction

Growth during the first few years of life plays an important role in setting body mass index (BMI) trajectories into childhood and adulthood [1, 2]. Once obesity is established, it is difficult to reverse [3]. In a recent Cochrane Review of interventions to prevent childhood obesity for children aged 0–5 years, there was moderate evidence from 16 randomised control trials that diet and exercise combined are effective on reducing zBMI scores, although these reductions were small [4]. For example, evidence from sixteen randomised control trials that combined diet and physical activity interventions for 0-5-year olds, compared with controls, reduced zBMI (mean difference −0.07 kg/m^2^, 95% confidence interval (CI) −0.14 to −0.01). There was little impact on zBMI for individual diet or physical activity interventions (Diet: mean difference −0.14, 95% CI −0.32 to 0.04; Physical activity; mean difference 0.01, 95% CI −0.10 to 0.13). A zBMI change of at least -0.25 for a clinically significant impact has been suggested [5].

While these studies do not account for the role of parental perception of their child’s weight, a recent meta-analysis exploring parental underestimates of child weight found that 50.7% (95% confidence interval 31.1%–70.2%) of parents underestimate their overweight/obese children’s weight [6]. Other studies have also reported similar findings [7–10]. The increase in children’s weight at a population level is clearly illustrated in a recent report, where the median child weight now falls on the 67^th^ centile rather than the 50^th^ centile (1990 baseline) [11].

Being overweight or obese in childhood is reported as a notable factor for being overweight or obese in adolescence and adulthood [12]. Overweight and obesity have an impact across the life course through the development of long term conditions and contributing to mental ill-health. The economic (personal and societal) costs are also immense [13].

When parents perceive childhood overweight and obesity as a concern, they also exhibit an inability to internalise this into their own lives [14]. Factors that contribute to parental failure to recognise or perceive overweight in their children are complex and multifaceted. They include parental beliefs and values of body weight, their own body weight [15], socio-economic factors [16], environmental factors [17] and societal normalisation of overweight [18, 19]. This normalisation of overweight and obesity within society is likely to be a contributing factor in the increase of childhood overweight and obesity [7, 19–21].

Effective, evidence-based interventions that focus on how best to support parents to understand their child’s growth and the influence the early years have on unhealthy weight gain is sparse [22, 23], with many studies and reports concluding that more research is needed [5, 24]. Growth charts enable children’s growth to be assessed by comparing them with a normal range for other children of the same age and gender, relative to a reference population. The serial measurements of height, weight and head circumference, taken as a child ages, are sensitive measures of their general health [24]. In many countries, growth charts are part of the parent-owned or parent-held child wellbeing books [25, 26] and used to provide access to understandable, evidence-based information to parents about their child’s growth compared to population norms [27]. Growth charts can play an important role in helping parents, at a glance, view the growth of their child. Despite this, parental understanding of the meaning of growth charts relative to their child(ren) is variable [28, 29].

However, some health professionals report that they often don’t use these charts with parents [30]. The reasons given include that parents have low health literacy [31]; parents find growth charts confusing [32] and misunderstand the meaning of chart percentiles (including the misperception that a high percentile is a sign of robustness), and confusion about population norms and how that applies to their child [28, 33–36]. Health professionals have also reported that the hand-held record book is often forgotten or lost, reducing its value for parents [37]. Notwithstanding this, parents do have a desire to hear if their child was at risk of obesity [38].

The rapid growth in mobile technology and in particular, the ubiquity of the smartphone is an opportunity to overcome such issues by digitising the hand-held growth record. A plethora of child growth labelled apps are available on the App Stores. Yet little evidence exists of the effectiveness of these apps in the prevention of childhood overweight and obesity [39].

### Child health in the New Zealand context

Child health in New Zealand is packaged as a comprehensive programme called Well-Child/Tamariki Ora. The Tamariki Ora/Well-Child programme is a comprehensive package of universal health services offered free to all New Zealand families/whānau for children from birth to 5 years. There are 13 planned health check contacts, with 11 checks provided between birth and 18-months. Several organisations specifically provide well-child services nationally, for example, Plunket (https://www.plunket.org.nz/) while other organisations are location-specific [40] or are Māori (indigenous population), providers. Services are provided by several health professions including midwifery, obstetrics, paediatrics, general practitioners and registered nurses. These services are often supported by kaiāwhina (community health workers) and vision and hearing technicians [26] and delivered in a variety of settings such as primary care settings, Marae (a place where for Māori communities gather and share) and the home. A Well Child/Tamariki Ora Health book is also produced and is given to all parents of new babies, to use as a hand-held record of health visits and immunisations. The Well Child / Tamariki Ora Health book also includes information for parents on milestones, safety and illnesses [41].

In this study, we aimed to co-create a child growth monitoring app and test its utility and feasibility. The focus on children from birth to two years was chosen because excessive weight gain in the first 100 weeks of life may be an important marker for the onset of overweight by school age and into adulthood [42, 43], and because of the high number of planned and free Well-Child/Tamariki Ora health visits in the first 24 months of life in New Zealand [26].

## Study objectives

This study aimed to co-create, develop and test the utility and feasibility of a smartphone-based digital child growth application (app) for use by New Zealand parents and caregivers with children aged 0–24 months.

## Methods

### Co-creation and development of the app

Between January—May 2018, an iterative process was used to gather detailed requirements for the app. This process involved a national on-line survey and focus groups of parents and/or caregivers with children under the age of 5 years, and interviews with health professionals (midwife, paediatrician, nurses) working in child health services. Recruitment processes for the survey used social media advertising (via FaceBook posts, Google Ads), using title tags such as “well-child providers”, and meta description tags such as “childhood development milestones.” Health provider and parent focus group participants were identified and invited to participate through existing networks.

Informed consent was obtained from all participants. Parent participants were asked what they knew about growth charts and the tools they used to help monitor their children’s growth. Health professionals were asked how they used growth charts with parents and barriers to their use. All participants were asked about what features and functions they would want in a digital growth chart tool. The final app features and functions would be pragmatically determined based on study time-frames and the technical difficulty and time need to build a particular feature.

To accompany the app and provide positive framed content and activity related prompts, within app notifications were used. App notifications are messages that display outside of the app and are used to communicate information (support knowledge development) and reminders (to act or do something) to the user. The type and content of the See How They Grow app (SHTG) notifications were underpinned by behaviour change theory [44, 45], and mHealth engagement research [46]. A programme logic was used to determine the type and frequency of these notifications.

In addition to routinely programmed information and activity notifications, specific action notification messages about entering a measurement were sent at 3-days and 14-days if no new measure had been added, and a third notification was sent if it was longer than 6-weeks since the last data entry. If participants had entered a measurement, they would not receive these message types.

The app was built for both Apple and Android operating systems using Ionic (https://ionicframework.com) and was made available for download from February–August 2019.

Ethics approvals were obtained from the Auckland University Human Participants Ethics Committee; Formative Study Reference 020166 and Feasibility Study Reference 022248. The feasibility study was registered on the Australia New Zealand Clinical Trials Registry (ANZCTR) reference ACTRN12619000905167.

### Feasibility and utility assessment

Participants were eligible to take part in the study if they were, 1) adults aged 18-years and over, 2) lived in NZ, 3) had a child or children under the age of 2-years, 4) had access and use of a smartphone capable of downloading the app and 5) were able to read and understand English. Eligible participants provided informed consent (agreement to complete a baseline and 12-week follow-up questionnaire, to enter at least three measures during the 12-week study period and for their app use data to be collected during and post-12-weeks (until the last participant had completed 12-weeks). All study procedures and data collection were managed through the app. Engagement with the app was measured using the date of last activity, frequency of use and self-reported feedback on utility, barriers, enablers and improvements (see Table 1). Underpinned by the broader app engagement literature, the engagement categories were explicitly designed for this study. The app had both off-line and on-line capability.

**Table 1:** Schedule of baseline and follow-up data collection.

Description	Baseline	12-weeks
Eligibility	✓	
E-informed consent	✓	
Age, sex, and ethnicity of adult users	✓	
Age and sex of the child	✓	
Home Region	✓	
Baseline Survey • *Demographics* • *Knowledge of growth charts* • *Use of growth charts*	✓	
Follow up Survey • *Reported changes in knowledge of growth charts* • *Reported changes in activities* • *Reported changes in foods offered* • *Perception of usefulness and utility* • *Barriers*, *enablers* • *Best features* • *Improvements*		✓
App engagement		
• Not engaged *No activity beyond day 1* • Somewhat engaged *No activity past 30 days* • Mostly Engaged *No activity past 60 days* • Actively Engaged *Activity throughout 90 days* • Very Engaged *Activity post 90 days*		✓
• *Frequency of app use*	Continuous throughout the study	
• *Type of app use*	Continuous throughout the study	
Feedback	At any time throughout the app	

### Recruitment

Recruitment promotion strategies included inviting participants from the co-creation phase, social media posts using keywords and meta tags such as “Māori well-child services”, “traditional Māori parenting” or child immunisations”; paid digital advertising, a study website, promotion through health provider networks and hard-copy flyers placed in new baby packs.

As a feasibility study, no sample size calculation was performed. All statistical analyses used SAS™ with descriptive statistics used to analyse participant characteristics, app engagement and utilisation variables and Chi-square and Fisher Exact Tests used to compare differences. We did not include invited users in these analyses due to the low numbers (n = 4). For analyses participants were grouped into age bands (18–19; 20–24; 25–29; 30–34; 35–39; 40–44, and 45–49).

## Results

### Co-creation and development of the See How They Grow app

One hundred and ten parents or caregivers responded to the requirements survey with 101 included in the final analyses. Nine participants were excluded as they provided no or limited information. The majority were NZ European ethnic group with only 8% reporting as Māori. Participants were between 20 and 39 years. Fifty-one per cent reported that their youngest child was ≤ 12-months. Of the four focus groups, three comprised mainly of Māori and Pasifika parents, while the fourth group was mainly composed of NZ European parents and caregivers. Focus group numbers ranged from 5 to 8 participants. Most participants were women. Face-to-face interviews involved two midwives, a well-child nurse educator and a paediatrician and seven other well-child health providers provided email comments.

All parents or caregivers reported that knowing how their children were growing was important. Discussions of their child’s growth needed to be grounded in the broader context of their culture and social situation. The influence of culture was intricately woven into how they, as parents make decisions about their child(ren). Therefore, any tool needs to have an element of understanding about the norms, values, ideas and behaviours that may be deeply rooted in a particular culture and form part of daily living, to be successful. Foods and activities that were culturally relevant, were suggested as simple ways to incorporate some cultural nuances into the SHTG app.

However, when growth was discussed in the context of mapping measures onto growth charts, focus group participants, and 72% (n = 73) of survey participants reported that they thought the growth charts were more a tool for the health professional than for them.

Health professionals all reported they used growth charts with the parents or caregivers they worked with, but that parental understanding of the purpose of growth charts was variable. Most noted that it was common for parents to forget to bring their hand-held book to the visit and consequently, the utility of the growth chart in the book was suboptimal due to a lack of measures entered.

All participant groups (parent and health professional) remarked that an electronic tool would be a useful addition to the well-child space. And that it was important that any digital tool helped to reinforce New Zealand-oriented information and evidence, and supported the relationships between parents and well-child providers and other health professionals, rather than be perceived as replacing them.

Parent or caregivers reported that they should be able to add their self-collected measures onto a digital chart. This capability elicited a cautionary reaction from the health professional participants, as they were concerned that parents might become obsessive about growth indicators as a singular measure of wellbeing. They also remarked that it would be important to convey that a self-measurement was different from one performed and recorded by a health professional and not confuse interpretations.

Three main themes emerged to describe barriers to digital tool use,

1. Functional (such as internet access, access to smart devices),
2. Acceptability and Utility (such as information relevancy, ease of use and cultural relevancy), and
3. Systems (e.g. privacy and security, information ownership).

### Determining and shaping the features and functions to include in the See How They Grow app

**Fig 1:**
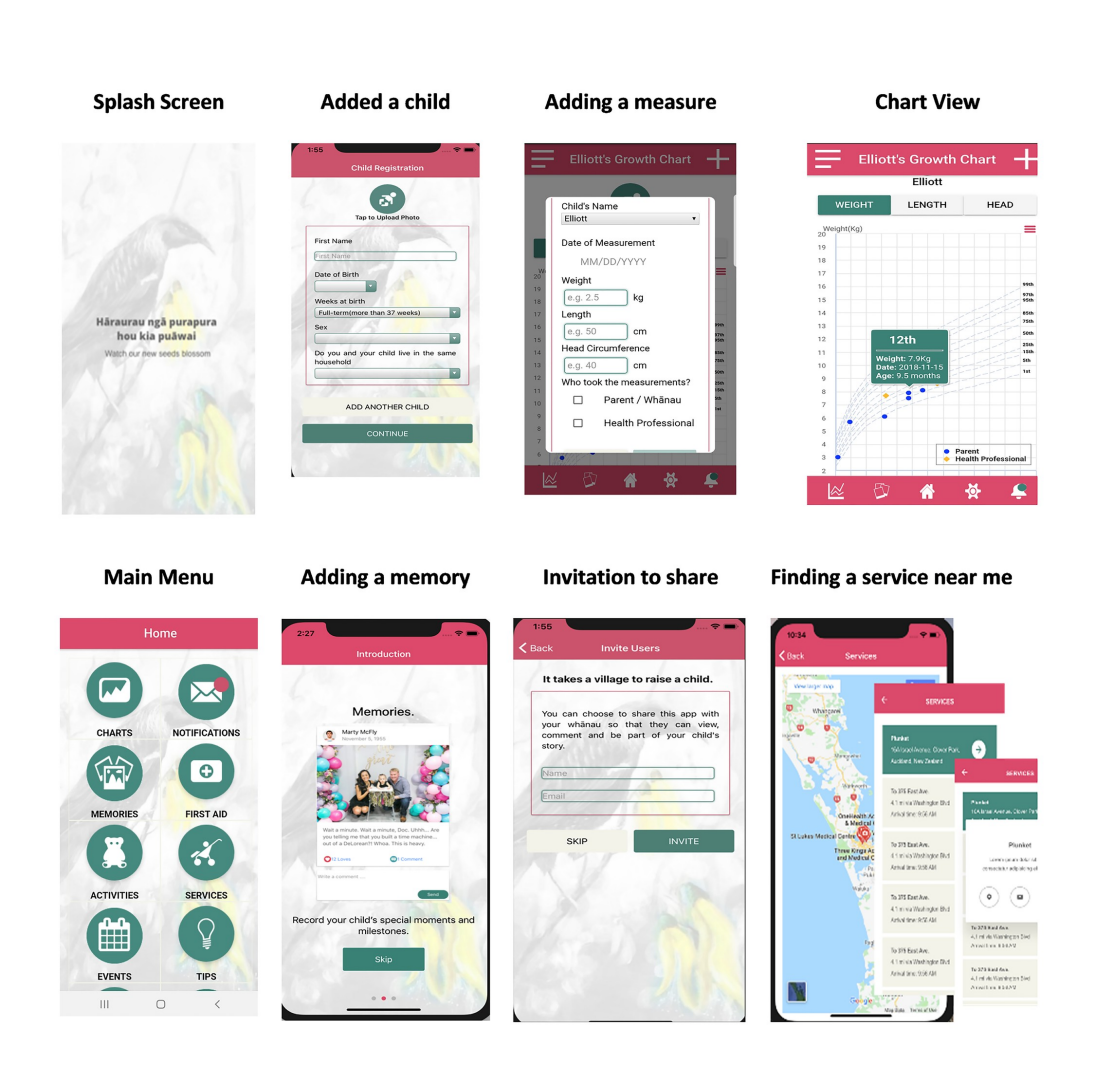
Images of some of the elements of the See How They Grow app.

Identified functions and features were converted into use-case stories, and these were mapped to underpinning conceptual knowledge and behavioural change themes. Table 2 presents the findings from this process, and Fig 1 provides a snapshot of images from the final SHTG app design. There were over 50 screens with which the participant could navigate and interact.

**Table 2:** See How They Grow app design features and functions.

Key Features Identified	Purpose/ Intent of Features and Behaviour Change	Conceptual Themes	Use Case Examples	Final Features and Functions adopted in See How They Grow
*“Ability to upload photos*.*”*	To share experiences, memories and have reinforcement (BCT 3.1)	Trust / Integrity	• To be able to add my comments or photos and have others (privately or publicly) view and comment so that it is more than just measures. • Create a longitudinal experience or history makes it difficult to stop interacting and difficult to delete, so I keep using it, and it is useful.	• Invite Others, • Create Memories, • Share Memories, • Enable others to like/comment on memories • Add photos
*“Share experiences*.*”*		Social / Cultural /Beliefs		
*“Share growth*.*”*				
*“Positive quotes/ comments—sometimes new mums just need to hear they are doing a good job*!*”*	To be rewarded, receive feedback, feel positive (BCT 10.3)	Independence / Empowerment	• To add or interact within the app and receive “unanticipated” rewards or appreciation that help reinforce behaviour • Having information in smaller sections and showing easy actions to promote a sense of capability and then skill development linked to my child(ren) age and stage of development • Messages (notifications) that pop-up when you complete an activity in the app such as a child’s measure or tick an immunisation with a positive message	• Informative and timely messages (notifications) • Likes and comments on Memories Page • Action prompts regarding measurements are about seeing a pattern and learning from each step, so if a change is considered important, then it seems more achievable.
		Emotional		
*“Clearer indication of what weight is healthy (e*.*g*. *percentile range) at what age*.*”*	Supports changes (BCT 2.2) Investment	Applicable Knowledge	• Real-time notification messages to reinforce activities and help to interpret or reinforce the activity • To have new or novel information presented either through news type feeds or notifications or links out if needed to read more. • Attracting the attention of the user is about relevance and supports the usefulness of the app • Supports idea for how to change	• Managing their child measures • Map measurements to their child and present back what that measure means. • Additional measurements entered prompts new information notifications • Resources and Tips sections,
*“See change and understand it*.*” “Child first aid*.*”*	New information and interpretation	Action		
*“Tips around keeping your child healthy*.*”*	Attention-grabbing			
*“Family support and* *“Family groups can be created*.*”* *“Self-entered data on other things*.*”* *“Reminders for important events*.*”*	Personalise	Skills / Managing Risk	• Being able to see data in a way that is relevant to me and minimises unhelpful/inappropriate comparisons • Creates achievements and motivations to keep me engaged • Integrated with my own calendar, reminders of important events/activities like immunisations, so I have it all in one place. • Can add other information important to me and my child(ren), like feeds or nappy change or sleep.	• Select avatars or add photos self and children. • Ability to create a whanau group to share the child(ren) journey and information • Calendar links to immunisation timelines. • Event entry within the app that links to the phone “native” calendar to minimise duplication and create synergy with common phone activity.
	Interpretation and relevancy			

Tables 3 and 4 present a sample of the notification message types sent to participants. The words in brackets such as [HI] or [FIRSTNAME] are tokens, and they are used to personalise the message. For example, the participant registered their First Name as Moana and ethnicity as Māori; therefore, the bracketed words would be replaced with Kia or Moana.

**Table 3:** Examples of the routine within app notifications.

**Routine messages using programme logic**	**Message Type**	**Timing (day)**	**Message content**
	Welcome	0	Welcome to the SHTG study. Over the next 3-months, you will be part of a study designed to test out our new app. Thank you for taking part.
	Welcome #2 regarding study contact details	2	[HI] [FIRSTNAME], thanks for taking part in the SHTG study. If you need to contact us, you can call us on 0800 3676444 or email us on seehowtheygrow@auckland.ac.nz
	Reminder about app functions–Resources	5	We hope you are enjoying the SHTG app. Did you know that the app has information about services and events relevant to your child as well as tips to support your child’s healthy growth?
	Reminder about app functions—immunisations & re flagging immunisations complete in the app	9	Has [CHILDNAME] had [HIS/HER] latest immunisations? If so, you can update this in the app. Go to immunisations and tick the ones [HE/SHE] has had.
	About growth tracking	10	Remember, a growth chart isn’t a test that children can pass or fail, and there isn’t a centile that he or she must reach to be healthy.

**Table 4:** Examples of data entry dependent messages.

**Data Entry Dependant Messages Examples**	**Message Description**	**Message content**	**Mapped to Conceptual Themes**
	First measure entered after registration	Thanks for entering in a weight measurement for [CHILDNAME]. It looks like [HE/SHE] is in the [CENTILE#] centile for weight.	Applicable Knowledge
	New measurement entered by professional	Thanks for entering in a new weight measurement for [CHILDNAME]. It looks like [HE/SHE] is continuing to grow along the same centile for weight which is great.	Applicable Knowledge
			Skills
	Decrease in weight (across one centile band) compared with the previous measure–Non-professional gathered measure	Thanks for entering in a new weight measurement for [CHILDNAME]. It looks like the rate that [HE/SHE] is growing might have decreased. This is usually ok, but it is a good idea to talk to your doctor or nurse if you are concerned. Also, look at TIPS for more information on centiles.	Applicable Knowledge
			Action
			Skills
	Message if there is a change across once centile bands up or down.	[HI], you can expect to see [CHILDNAME] growth line stay roughly in the same area of the chart as [HE/SHE] grows, but it probably won’t follow a centile line exactly. It’s perfectly normal for [HIS/HER] growth line to move between centiles occasionally.	Applicable Knowledge
			Skills
			Managing Risk

### Feasibility study

A total of 225 people from across NZ downloaded the app. Of these, 17 (7.5%) were excluded as no data were entered, and 208 (92.4%) participants were eligible. Of the eligible participants, eight (4%) registered two children, while all others registered one child. Of the 208 eligible participants, 101 (49.5%) completed the follow-up survey (Table 5). The majority of participants (191, 92%) reported that they were female (*P =* 0.008) and the mother of the child (193, 93%); were mostly European (78%). Māori participants were significantly less likely to complete the follow-up survey (baseline 14% were Maori, Follow-up 8%: *p =* 0.010). There was no difference in participants by age at baseline and follow up were similar (*p* 0.227*)*. At baseline, the mean child age was 7.8 months (median 5.7 months, SD 6.4 months). There were more participants with children aged ≤ 6-months completing the follow-up survey compared with those aged ≥ 6-months (*p* 0.001).

**Table 5:** Characteristics of participants at baseline and follow-up.

	Baseline		12-week Follow up Survey Completed		
	N	%	N	%	Chisq p *
Total	208	-	101	-	
Relationship to child					
Father	13	6	4	4	
Friend	1	0	0		
Mother	193	93	98	95	
Uncle	1	0	1	1	
Ethnicity (note more than one can be selected)					
European	163	78	84	82	0.337
Maori	30	14	8	8	0.010
Asian	7	3	5	5	
Pacific	11	5	4	4	
Chinese	6	3	5	5	
Indian	9	4	3	3	
Other	16	8	8	8	
Gender					0.008
Female	191	92	98	95	
Male	17	8	5	5	
Collapsed Age Groups					0.227
18–19	3	1	2	2	
20–24	16	8	9	9	
25–29	53	25	25	24	
30–34	65	31	38	37	
35–39	61	29	26	25	
40–44	9	4	3	3	
45–49	1	0	0		
Region					
Auckland	97	47	52	50	
Bay of Plenty	13	6	8	8	
Canterbury	13	6	8	8	
Hawke’s Bay	3	1	2	2	
Manawatu-Wanganui	6	3	3	3	
Northland	21	10	8	8	
Otago	4	2	1	1	
Southland	2	1	0		
Taranaki	2	1	2	2	
Tasman	1	0	1	1	
Waikato	21	10	5	5	
Wellington	23	11	12	12	
West Coast	2	1	1	1	

*Chi-squared P value comparing participants that completed the follow-up survey to those that didn’t.

### Measurement data entered

Almost half (100; 48%) entered measures (weight, height, head circumference) on three occasions or more, with 25% (n = 52) adding two measurements. Weight and height were the most frequently entered of all measure types (70% and 66% respectively). Ninety per cent of all measurements were recorded as being from a Health Professional with 70% of these being for children ≤ 6-months.

### Engagement

#### Date of last activity

Five categories from not engaged (no activity after day one) to very engaged (activity ongoing after 12-weeks) were used to categorise participant app use data. Forty-four per cent (n = 91/208) of baseline participants were engaged up to or over the 12-weeks. This group were more likely to complete the 12-week survey. There were no differences in the ages of participants and their last activity (Chi-squared *p =* 0.494). However, participants who identified as Māori were more likely to have no app activity recorded after 60 days (Fisher Exact Test *p =* 0.017) compared to Non-Māori (Table 6).

**Table 6:** Participant last app activity by engagement category.

			Baseline		12-week Follow-up	
			N	%	N	%
	Engagement Categories	Total	208	-	101	
1	Not engaged	No activity beyond day 1	48	23	8	8
2	Somewhat engaged	No activity past 30 days	38	18	6	6
3	Mostly Engaged	No activity past 60 days	31	15	15	15
4	Actively Engaged	Activity up to 90 days	25	12	17	17
5	Very Engaged	Activity past 90 days	66	32	55	54

#### Navigation and exploring screens

We found no difference in the number of screens navigated to and viewed, irrespective of when the last app activity was (see Table 7).

**Table 7:** The number of screen views by last activity engagement categories.

				Screen Views						
Engagement Category			N (%)	mean	sd	median	lower IQR*	upper IQR*	min	max
1	Not engaged	No activity beyond day 1	48 (23)	22.3	12.7	20	12	34	2	47
2	Somewhat engaged	No activity past 30 days	38 (18)	32	32.5	23.5	17.5	30.5	13	141
3	Mostly Engaged	No activity past 60 days	31 (15)	22.8	22.6	15	8	34.5	2	90
4	Actively Engaged	Activity up to 90 days	25 (12)	14.1	8.9	13	9	17	1	41
5	Very Engaged	Activity past 90 days	66 (32)	33.5	29.6	28	15	41	2	138

*Interquartile range

#### The role of notifications in supporting app activity and engagement

**Fig 2:**
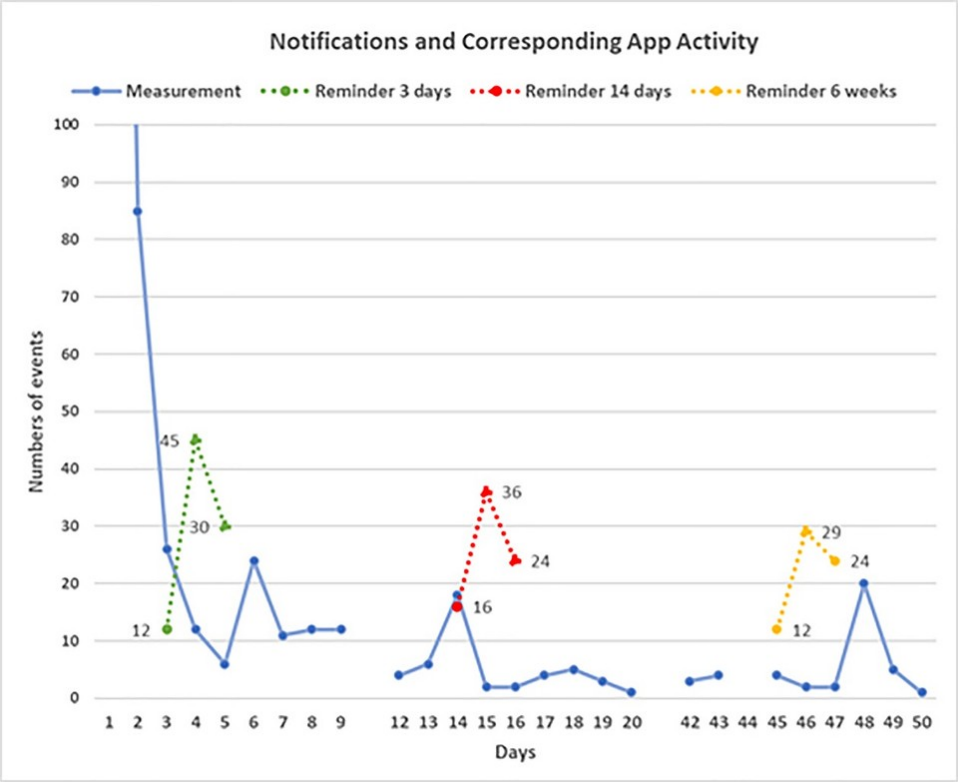
Notifications and corresponding app activity.

Measurements entered after a reminder notification was received, are presented in Fig 2. The day three reminders were sent to 87 participants prompting 42 new measurements entered on the same day or the next. The day 14 reminder was sent to 76 participants, and 24 new measurements were added. The week six reminder was sent to 65 participants, and 28 new measurements were entered.

### Improved growth chart interpretation and understanding

At baseline, the majority of participants responded that they were aware of the Well Child Tamariki Ora book (201; 97%) and 96% (192) reported finding them useful for tracking their child’s growth. One-third reported that they understood the information conveyed by growth charts only "a little" (68, 34%) while 65% (130) reported understanding them clearly and 1% reporting not understanding them at all. The majority of participants (173; 86%) reported that the growth charts were relevant to their child. Of the 101 (48.5%) participants who completed the 12-week survey, 72 (70%) agreed that the app helped them to understand and interpret growth charts better. All except one participant reported that they found that the visual presentation and the interpretive information provided after measurements were entered were informative and relevant to their child. The following quotes illustrate how some participants found the app useful:

*It gives the caregiver a visual representation to see how their child is growing, and it sends useful notifications*.

    Parent of under 6-month child

*Because we could show the GP + the paediatrician when we had to see them*. *The DHB* [District Health Board] *Paediatrician had paper and had to replicate the plots*, *and the GP only entered the measurement she took*, *which didn’t show a trend*. *We had the birth weight*, *plunket weights*, *nurse weights*, *paediatrician AND GP weights on the graph*.

    Parent of under 6-month child

Four participants reported that they did not find SHTG useful overall. *"Potential privacy issues"* was the only free-text comment documented to explain this response.

Twenty-one per cent (22) reported that they had learnt something new at the end of the 12-week study. Of these, 19 were ≤ 34-years of age, and 17 had children ≤ 6-months of age, with the majority of responses being from European ethnicity (18). Box 1 presents the main themes where participants reported gaining new knowledge or understanding.

**Box 1. What was something new that I learnt while using the SHTG app?**

**Table:** 

**Knowledge Related to their Child**	**Understanding Centiles**	**General Knowledge of Growth Charts**
• How well they are growing (hopefully) and when to be concerned • I read extra about growth charts to feel better than my child was dropping percentile points. I spoke with my Plunket nurse who reassured me that my child’s growth, while not following the line, was still tracking very well • If my son is on average growth. • That it’s not a test that a child must pass. Each child is different and they don’t have to be a certain centile to be healthy • That a general consistent growth is healthy! And I would imagine it would be really good to notice weight loss due to illness or allergy • It was interesting to see where his different measurements sat on the percentile • When to be concerned & contact a health professional • That so long as your child follows their own line and keeps making progress they are on the right track	• What the percentiles meant • Differences in percentiles • How the percentiles work • Why they are relevant • The way percentiles work	• How Plunket nurses use a growth chart • That it’s an accumulation of the data set/trend of growth that matters not a single point that really matters. • That they just have to follow one line not try to get to 100% • That growth charts include height and head as well as weight • The data used to formulate the growth charts • I really liked the blub below the chart explaining that all babies grow differently etc

### Food and activity knowledge and behaviour changes

There were 81 eligible participants after excluding responses from parents who signalled that they were still exclusively breastfeeding (17/101) and those that did not respond to this question (3). Of these 81 participants, the majority (71) reported: "No—SHTG did not change what foods they provided," whereas 10 participants reported either "yes" (n = 6) or "a little" (n = 4) to making a change to the foods they offered. “*Offered more variety”*, “*amount of food offered”*, and “*new ideas of what to offer”* were the typical responses.

Eleven participants reported "yes" (n = 8) or "a little" (n = 3) to increasing the activities they and their child were doing. The remaining participants either did not answer this question (n = 13) or reported No (n = 77). "*More tummy time*" and "*more playtime*" were the common free-text responses with one participant remarking that SHTG "*Helped me figure out more simple yet effective activities to do with my baby"*.

### Other features and functions used

Sharing the app was highlighted as an important feature in the co-creation phase, and 41 (20%) participants sent a share app invitation. All were parents or caregivers of children aged under 12-months. Being the Partner of the invitee, was the main category for whom was invited (n = 17), followed by Grandparents (n = 3), Sisters/Brothers, Aunties/Uncles, extended whānau/family and Other, were all equally mentioned (n = 2 respectively). Only three people accepted the invitation. Despite the low use of this feature, 74% (n = 76) of survey respondents, reported that this is an important feature.

Adding self-measurements and reported positively by 95%, followed by the resources and tips screens (40%). The link to services and activities and adding photos were the third and fourth liked features, 17% and 12% respectively. Interestingly, 23% liked the notification messages while almost the same proportion (25%) did not. This latter group were more likely to have their last activity recorded at 0–30 days, whereas the former group were actively using the app for the full 12-weeks.

### Technical issues

Sixty-two per cent of participants (n = 64) reported that they had no technical problems with the app. Not being able to delete a measure or zoom in on the charts were reported as problems, albeit these capabilities had not been designed into the app.

### Acceptability feedback and new features

The majority of participants (87%; 88) reported that they would recommend the app to others. One hundred participants (97%) said that the app was culturally appropriate and that the role of culture needed to be woven into the information provided, as illustrated in the following response.

*There are lots of cultural difference for Chinese mums during the first month after the birth of baby, including food, diet, beliefs*.

    Parent of under 6-month child

The majority (86%, 87) recommended that the app should include monitoring for children over 24-months old.

While participant comments overall were positive, some participants (7) did not find the app useful, appealing or easy to use for example one participant’s remarked,

The app is just a bit clunky to use. If it was easier to operate or automatically updated, then the app would be awesome!

    Parent with a 6-12-month child.

A range of suggestions for new features and general app improvements are illustrated in Box 2.

**Box 2. New features and functions**

**Table:** 

• *Having tick boxes for when immunisations are completed*.
• *Being able to zoom into the graphs*.
• *Having multimedia options such as videos on what to expect at milestones*.
• *Being able to delete incorrect entries easier*.
• *Feed and sleep tracking*.
• *More ideas for activities*.
• *Have more information on how to stimulate motor development and cognitive abilities*.

Finally, the importance of being able to share the information with a family doctor or health provider was highlighted, with one participant commenting on the importance of this feature for them.

*Ability to email to GP* [General Practitioner], *so the export says where the data is from and its provenance*. *I had to explain to the GP who had entered the data incorrectly into her MedTech chart*, *and said ’growth looks normal’ THEN I showed her the app and my growth chart and then my interpretation and she suddenly realised she’d inputted her data incorrectly*.

    Parent with a 6-12-month child.

## Discussion

The findings from this feasibility study highlight that the digitalisation of growth charts and embedding these within a mobile app is an acceptable and potentially preferable modality for parents and carers to capture and monitor their child’s growth. This finding was most apparent amongst parents with children under 6- months. While not a panacea, increasingly research is finding that a variety of interventions in the prenatal and early infancy period. Findings reported were improvement in positive parental health practises, such as longer breastfeeding, later introduction of solids and increased child activity, all having a positive impact on early obesity compared to control groups [23].

The lower engagement by Māori compared to non-Māori suggests that SHTG was missing something that could have kept this cohort engaged longer. The role of culture in child-rearing and perceptions of growth and development needs to be better understood and interventions designed that account for these differences, otherwise, it is likely they could increase the disparity [47–49].

The majority of app activity was related to entering measures. When measures were entered, the data were overwhelmingly labelled as being from health professionals; reinforcing the importance parents placed on this trusted information. Importantly, we found knowledge and understanding of growth measures increased over the study period. Still, the impact for Māori was muted due to the lower SHTG engagement over time, similarly for Pasifika populations who were under-represented as participants overall; despite these groups being well represented in the co-creation phase. Future research which includes interviews or focus groups with participants at the end of these feasibility studies, would help provide more contextualisation and understanding of the usage data than survey tools can.

The impact of personalised and tailored messages have been well researched for text messaging interventions [49–51]. Similar in principle to text messaging are app notification messages. The added advantage of app notification messages is that they can also guide the end-user to further information within the app or to record an activity. While text messaging can also direct users to further information, it is difficult to measure unless participants actively report their behaviour. With app notifications, measuring responses is much simpler, as the receipt of the notification and response can be captured and counted. We found that for some, the notification feature was annoying, but some notifications did trigger the corresponding activity suggesting that they can be useful tools to engage end-users. Other studies have found similar outcomes, for example, Freyne et al. [52] report in their study on partial meal replacement that participants with access to self-monitoring tools and notifications accessed the app more than those with program information only. They also report that activity in the app was almost double around the time(s) notifications were sent compared to other times.

There was no notable impact on any behaviour changes regarding nutrition or activity by participants that may have been influenced by using the app. However, the positive direction of notification and action suggests that further development of the app to include a comprehensive adaptive and responsive element underpinned by theory could influence both knowledge and behaviour.

Further understanding of the reasons for family or friends not accepting invites to use the app, is needed. The main areas for improvement were minor functional improvements and some feature enhancements suggesting that the co-creation phase had identified many of the desirable features and functions. Furthermore, the additional features suggested were not unexpected, for example, to expand it to encompass a more extensive age range, at minimum 0–5 five-year-olds, and to have more interactive elements and tailored age-appropriate multi-media tools. These latter two attributes are commonly available in more commercially available applications (for example in travel and banking mobile applications).

The low Māori and Pasifika engagement suggests that exploring the cultural aspects and contextual understandings of parenting are essential to ensure that the final product supports and encourages engagement beyond the first few interactions.

Overall, the SHTG feasibility study found that a digital growth chart and mHealth intervention has the potential to be acceptable and useful for supporting parents in their knowledge of growth charts and by encouraging them to actively monitor their child’s growth. This finding aligns with other results that have used digital tools in the prevention of obesity in older children. While not specific to the use of growth charts, a systematic review of studies using wireless and mobile technologies to prevent and treat paediatric obesity, report that several of the studies included in the review describe increases in physical activity, increased fruit and vegetable intake and improved self-monitoring [39].

## Conclusion

The See How They Grow app was found to be acceptable, feasible and easy to use by the majority of the participants. While this study was not powered to detect changes or to measure the impact of the app on knowledge and behaviour change, the findings suggest that further development is worth pursuing. Despite high participation in the co-creation phase, the low uptake of indigenous and Pasifika populations is a notable limitation to the findings. Critical next steps will be to include more depth, culturally appropriate and relevant content to meet the diverse needs of all population groups. Similarly, understanding the reasons for the higher disengagement amongst some population groups is also needed. This should be followed by more research that explores the impact of digital growth and mHealth tools on reducing childhood overweight and obesity.

## Supporting information

S1 File (PDF)

## References

[1] Denney-Wilson E, Laws R, Russell CG, Ong KL, Taki S, Elliot R, et al Preventing obesity in infants: the Growing healthy feasibility trial protocol. BMJ Open. 2015;5(11):e009258 10.1136/bmjopen-2015-009258 26621519 PMC4679836

[2] Pryor LE, Tremblay RE, Boivin M, Touchette E, Dubois L, Genolini C, et al Developmental Trajectories of Body Mass Index in Early Childhood and Their Risk Factors: An 8-Year Longitudinal Study. JAMA Pediatrics. 2011;165(10):906–12. 10.1001/archpediatrics.2011.153 21969392

[3] Mead E, Brown T, Rees K, Azevedo LB, Whittaker V, Jones D, et al Diet, physical activity and behavioural interventions for the treatment of overweight or obese children from the age of 6 to 11 years. Cochrane Database of Systematic Reviews. 2017(6). 10.1002/14651858.CD012651 28639319 PMC6481885

[4] Brown T, Moore THM, Hooper L, Gao Y, Zayegh A, Ijaz S, et al Interventions for preventing obesity in children. Cochrane Database of Systematic Reviews. 2019(7). 10.1002/14651858.CD001871.pub4 31332776 PMC6646867

[5] Hankey C, editor. Advanced Nutrition and Dietetics in Obesity: John Wiley & Sons Ltd.; 2018.

[6] Lundahl A, Kidwell KM, Nelson TD. Parental Underestimates of Child Weight: A Meta-analysis. Pediatrics. 2014;133(3):e689–e703. 10.1542/peds.2013-2690 24488736

[7] Jeffery AN, Metcalf BS, Hosking J, Mostazir MB, Voss LD, Wilkin TJ. Awareness of body weight by mothers and their children: repeated measures in a single cohort (EarlyBird 64). Child Care Health Dev. 2015;41(3):434–42. 10.1111/cch.12167 24912623

[8] Musaad SMA, Donovan SM, Fiese BH. Parental perception of child weight in the first two years-of-life: a potential link between infant feeding and preschoolers’ diet. Appetite. 2015;91:90–100. 10.1016/j.appet.2015.03.029 25843938

[9] McKee C, Long L, Southward LH, Walker B, McCown J. The Role of Parental Misperception of Child’s Body Weight in Childhood Obesity. Journal of Pediatric Nursing. 2016;31(2):196–203. 10.1016/j.pedn.2015.10.003 26521022

[10] Manios Y, Moschonis G, Karatzi K, Androutsos O, Chinapaw M, Moreno LA, et al Large proportions of overweight and obese children, as well as their parents, underestimate children’s weight status across Europe. The ENERGY (EuropeaN Energy balance Research to prevent excessive weight Gain among Youth) project. Public Health Nutrition. 2015;18(12):2183–90. 10.1017/S136898001400305X 25650819 PMC10271737

[11] Public Health England. Patterns and trends in child obesity: a presentation of the latest data on child obesity. 2017. https://www.slideshare.net/PublicHealthEngland/patterns-and-trends-inchild-obesity-june-2017.

[12] de Onis M, Blossner M, Borghi E. Global prevalence and trends of overweight and obesity among preschool children. American Journal of Clinical Nutrition. 2010;92(5):1257–64. 10.3945/ajcn.2010.29786 20861173

[13] Malik VS, Willett WC, Hu FB. Global obesity: trends, risk factors and policy implications. Nat Rev Endocrinol. 2013;9. 10.1038/nrendo.2012.199 23165161

[14] Appleton J, Fowler C, Brown N. Parents’ views on childhood obesity: qualitative analysis of discussion board postings. Contemporary Nurse. 2017;53(4):410–20. 10.1080/10376178.2017.1358650 28728473

[15] Wang Y, Min J, Khuri J, Li M. A Systematic Examination of the Association between Parental and Child Obesity across Countries. Adv Nutr. 2017;8(3):436–48. 10.3945/an.116.013235 28507009 PMC5421118

[16] Templin T, Cravo Oliveira Hashiguchi T, Thomson B, Dieleman J, Bendavid E. The overweight and obesity transition from the wealthy to the poor in low- and middle-income countries: A survey of household data from 103 countries. PLOS Medicine. 2019;16(11):e1002968 10.1371/journal.pmed.1002968 31774821 PMC6880978

[17] Nnyanzi LA, Summerbell CD, Ells L, Shucksmith J. Parental response to a letter reporting child overweight measured as part of a routine national programme in England: results from interviews with parents. BMC public health. 2016;16:846–. 10.1186/s12889-016-3481-3 27544538 PMC4992560

[18] Miller JC, Grant AM, Drummond BF, Williams SM, Taylor RW, Goulding A. DXA Measurements Confirm that Parental Perceptions of Elevated Adiposity in Young Children are Poor. Obesity. 2007;15(1):165–. 10.1038/oby.2007.558 17228044

[19] Burke MA, Heiland FW, Nadler CM. From “Overweight” to “About Right”: Evidence of a Generational Shift in Body Weight Norms. Obesity. 2010;18(6):1226–34. 10.1038/oby.2009.369 19875997

[20] Parkinson KN, Reilly JJ, Basterfield L, Reilly JK, Janssen X, Jones AR, et al Mothers’ perceptions of child weight status and the subsequent weight gain of their children: a population-based longitudinal study. International Journal of Obesity. 2017;41(5):801–6. 10.1038/ijo.2017.20 28119532 PMC5418556

[21] Pryke R. Childhood obesity: running from this crisis of ‘normalisation’ won’t work. British Journal of General Practice. 2018;68(673):358–9. 10.3399/bjgp18X698009 30049753 PMC6058640

[22] Baur LA, Garnett SP. Early childhood—a critical period for obesity prevention. Nature Reviews Endocrinology. 2019;15(1):5–6. 10.1038/s41574-018-0131-0 30446743

[23] Blake-Lamb TL, Locks LM, Perkins ME, Woo Baidal JA, Cheng ER, Taveras EM. Interventions for Childhood Obesity in the First 1,000 Days A Systematic Review. American Journal of Preventive Medicine. 2016;50(6):780–9. 10.1016/j.amepre.2015.11.010 26916260 PMC5207495

[24] World Health Organisation. Report of the Commission on Ending Childhood Obesity. WHO; 2017. Contract No.: WHO/NMH/PND/ECHO/17.1.

[25] Webster J, Forbes K, Foster S, Thomas I, Griffin A, Timms H. Sharing Antenatal Care: Client Satisfaction and Use of the ‘Patient-held Record’. Australian and New Zealand Journal of Obstetrics and Gynaecology. 1996;36(1):11–4. 10.1111/j.1479-828x.1996.tb02912.x 8775241

[26] Ministry of Health. Well Child Tamariki Ora visits 2015 [updated 30 June 2015. Available from: https://www.health.govt.nz/your-health/pregnancy-and-kids/services-and-support-you-and-your-child/well-child-tamariki-ora-visits.

[27] TechNet-21. TechNet-21 https://www.technet-21.org/en2019 [Available from: https://www.technet-21.org/en/topics/home-base-records.

[28] Ben-Joseph EP, Dowshen SA, Izenberg N. Do Parents Understand Growth Charts? A National, Internet-Based Survey. Pediatrics. 2009;124(4):1100–9. 10.1542/peds.2008-0797 19786446

[29] Ansari Z. How to Design Effective Child Growth Apps, Not Just Attractive Ones: Evaluating the design of child growth chart apps and investigating end-user design preferences and effective design for an app-based child growth chart: Masters Thesis: University of Auckland; 2018.

[30] Lakshman R, Landsbaugh JR, Schiff A, Cohn S, Griffin S, Ong KK. Developing a programme for healthy growth and nutrition during infancy: understanding user perspectives. Child Care Hlth Dev. 2012;38(5):675–82. 10.1111/j.1365-2214.2011.01283.x 21752063

[31] Kickbusch I, Walt S, Maog D. Navigating Health: The role of health literacy. 2005 [Available from: http://www.ilonakickbusch.com/health-literacy/index.shtml.

[32] Sachs M, Sharp L, Bedford H, Wright CM. ’Now I understand’: consulting parents on chart design and parental information for the UK-WHO child growth charts. Child Care Hlth Dev. 2012;38(3):435–40. 10.1111/j.1365-2214.2011.01256.x 21668464

[33] Laraway KA, Birch LL, Shaffer ML, Paul IM. Parent perception of healthy infant and toddler growth. Clin Pediatr. 2010;49(4):343–9. 10.1177/0009922809343717 19745095 PMC3623679

[34] Valencia AC, Thomson CA, Duncan B, Arthur A. Evaluating Latino WIC Mothers’ Perceptions of Infant’s Healthy Growth: A Formative Assessment. Matern Child Hlth J. 2016;20(3):525–33. 10.1007/s10995-015-1850-7 26530036

[35] Ben-Joseph EP, Dowshen SA, Izenberg N. Public understanding of growth charts: A review of the literature. Patient Educ Couns. 2007;65(3):288–95. 10.1016/j.pec.2006.09.001 17081719

[36] Jones AR, Parkinson KN, Drewett RF, Hyland RM, Pearce MS, Adamson AJ, et al Parental perceptions of weight status in children: the Gateshead Millennium Study. International journal of obesity (2005). 2011;35(7):953–62. 10.1038/ijo.2011.106 21673651 PMC3154641

[37] World Health Organisation. WHO recommendations on home-based records for maternal, newborn and child health Geneva: World Health Organization; 2018. 30325618

[38] Butler ÉM, Derraik JGB, Glover M, Morton SMB, Tautolo E-S, Taylor RW, et al Acceptability of early childhood obesity prediction models to New Zealand families. PLOS ONE. 2019;14(12):e0225212 10.1371/journal.pone.0225212 31790443 PMC6886750

[39] Turner T, Spruijt-Metz D, Wen CKF, Hingle MD. Prevention and treatment of pediatric obesity using mobile and wireless technologies: a systematic review. Pediatric Obesity. 2015;10(6):403–9. 10.1111/ijpo.12002 25641770 PMC4499498

[40] Ministry of Health. Find a Well Child Tamariki Ora provider https://www.health.govt.nz/your-health/pregnancy-and-kids/services-and-support-you-and-your-child/well-child-tamariki-ora-visits/find-well-child-tamariki-ora-provider2020 [updated 29 September 2016 Available from: https://www.health.govt.nz/your-health/pregnancy-and-kids/services-and-support-you-and-your-child/well-child-tamariki-ora-visits/find-well-child-tamariki-ora-provider.

[41] Ministry of Health. Well Child Tamariki Ora My Health Book. https://www.healthed.govt.nz/system/files/resource-files/HE7012_Well%20Child%20Tamariki%20Ora_0.pdf: Ministry of Health 2010 (revised 2019).

[42] Glavin K, Roelants M, Strand BH, Júlíusson PB, Lie KK, Helseth S, et al Important periods of weight development in childhood: a population-based longitudinal study. BMC Public Health. 2014;14(1):160 10.1186/1471-2458-14-160 24524269 PMC3925776

[43] Baird J, Fisher D, Lucas P, Kleijnen J, Roberts H, Law C. Being big or growing fast: systematic review of size and growth in infancy and later obesity. BMJ. 2005;331(7522):929 10.1136/bmj.38586.411273.E0 16227306 PMC1261184

[44] Michie S, Ashford S, Sniehotta F, Dombrowski S, Bishop A, French D. A refined taxonomy of behaviour change techniques to help people change their physical activity and healthy eating behaviours: the CALO-RE taxonomy. Psychol Health. 2011;26:1479–98. 10.1080/08870446.2010.540664 21678185

[45] Michie S, West R, Campbell R, Brown J, Gainford H. ABC of behaviour change theories. Great Britain: Silverback Publishing 2014.

[46] Taki S, Lymer S, Russell CG, Campbell K, Laws R, Ong KL, et al Assessing User Engagement of an mHealth Intervention: Development and Implementation of the Growing Healthy App Engagement Index. JMIR Mhealth Uhealth. 2017;5(6):e89 10.2196/mhealth.7236 28663164 PMC5509951

[47] Shackleton N, Derraik JGB, Audas R, Taylor RW, Glover M, Morton SMB, et al Decomposing ethnic differences in body mass index and obesity rates among New Zealand pre-schoolers. International Journal of Obesity. 2019;43(10):1951–60. 10.1038/s41366-019-0390-4 31197250

[48] Glover M, Wong SF, Fa’alili-Fidow J, Derraik JGB, Taylor RW, Morton SMB, et al Ranked Importance of Childhood Obesity Determinants: Parents’ Views across Ethnicities in New Zealand. Nutrients. 2019;11(9):2145 10.3390/nu11092145 31500336 PMC6769712

[49] Dobson R, Whittaker R, Bartley H, Connor A, Chen R, Ross M, et al Development of a Culturally Tailored Text Message Maternal Health Program: TextMATCH. JMIR Mhealth Uhealth. 2017;5(4):e49 10.2196/mhealth.7205 28428159 PMC5418521

[50] Abroms LC, Whittaker R, Free C, Mendel Van Alstyne J, Schindler-Ruwisch JM. Developing and Pretesting a Text Messaging Program for Health Behavior Change: Recommended Steps. JMIR Mhealth Uhealth. 2015;3(4):e107 10.2196/mhealth.4917 26690917 PMC4704898

[51] Gallegos D, Russell-Bennett R, Previte J, Parkinson J. Can a text message a week improve breastfeeding? Bmc Pregnancy and Childbirth. 2014;14 10.1186/s12884-014-0374-2 25369808 PMC4237760

[52] Freyne J, Yin J, Brindal E, Hendrie GA, Berkovsky S, Noakes M. Push Notifications in Diet Apps: Influencing Engagement Times and Tasks. International Journal of Human–Computer Interaction. 2017.

