# Supplementary material for: See How They Grow: Testing the feasibility of a mobile app to support parents’ understanding of child growth charts
Source: PLoS One. 2021 Feb 19;16(2):e0246045. doi: 10.1371/journal.pone.0246045 (PMC7894826; doi:10.1371/journal.pone.0246045)

Project Detail
Name: See How They Grow
Study Type: Feasibility Study on Child Grow Chart Mobile App
Product: Mobile app
Platform: iOS and Android
Coding: IONIC
Timeline:

Task	Week 1 (25/06)	Week 2 (02/07)	Week 3 (09/07)	Week 4 (16/07)	Week 5 (23/07)	Week 6 (30/07)	Week 7 06/08)	Week 8 (13/08)	Week 9 (20/08)	Week 10 (27/08)	Week 11 (03/09)	Week 12 (10/09)	Week 13 (17/09)	Week 14 (24/09)
Timeline development														
Flow digram														
Confirm flow diagram / UX deisgn														
Content development														
UI and graphic design														
Coding / Programming process														

App Usage Senario / Flow Diagram

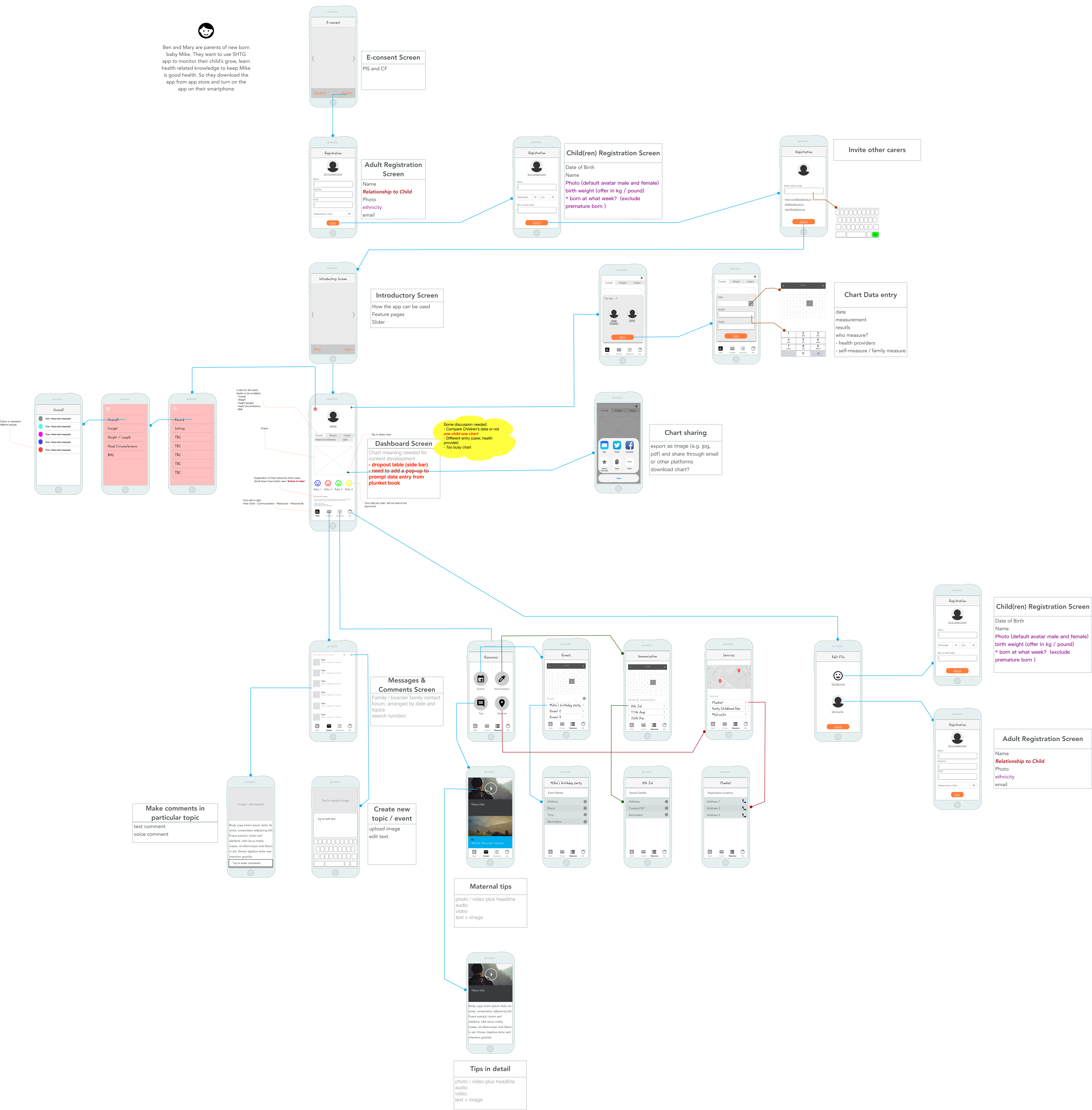

Supplement: S1 File — (PDF) [file pone.0246045.s001.pdf]
